# “Current training” in Cardiac devices – a Cardiology trainee perspective: a questionnaire survey

**DOI:** 10.15694/mep.2017.000130

**Published:** 2017-07-17

**Authors:** Cheuk Wing Jonathan Lee, Archana Rao

**Affiliations:** 1 Arrow Park Hospital; 2 Liverpool Heart and Chest Hospital

**Keywords:** “Current training”, Cardiac devices, Trainee perspective, cardiac devices training

## Abstract

This article was migrated. The article was marked as recommended.

A national survey (questionnaire) on Cardiology trainees undertaken on behalf of the author by the British Junior Cardiologists Association (BJCA) on training opportunities, needs, attitudes and perceived barriers that exist within “current training” in cardiac devices suggests lack of opportunity for hands on training and poor attitude to training resulting in a perceived lack of competence. The conflict between service commitment and training was a recurring theme.

## Introduction

Medical education’s ultimate aim is to create and nurture a skilled, knowledgeable and up to date cadre of professionals who maintain and develop their expertise over a life-long career ( Swanwick &Buckley 2010).

The Francis Report (2013) affirmed that the patient must be the first priority of the NHS by ensuring that, within available resources, they received effective care from caring, compassionate and committed staff. It stated that education should focus on the practical requirements of delivering compassionate care.

In an ever increasing competitive and litigious environment this is no mean task given the on-going challenges posed by concerns over patient safety, regulatory requirements, increasing medical student numbers and accelerated patient through put.

Work and time pressures, staff availability and patient expectation further impacted on training and resulted in poor morale within the junior doctors.

This has resulted in problems retaining trainees throughout the training pathway until they acquire the evidence of completion of training and progress required to fill consultant vacancies ( Hughes 2013).

This is particularly true of difficulties encountered in practical specialities (such as interventional cardiology) where in procedure based skill competencies were traditionally developed by transitioning gradually from observing to independently performing these procedures.

The author is an Interventional Cardiologist by profession and is actively involved in training and education of the medics at all stages and believes that it would be useful for the reader to establish context with regard to stages in the graduate and post graduate training of medics.

**Fig 1.**
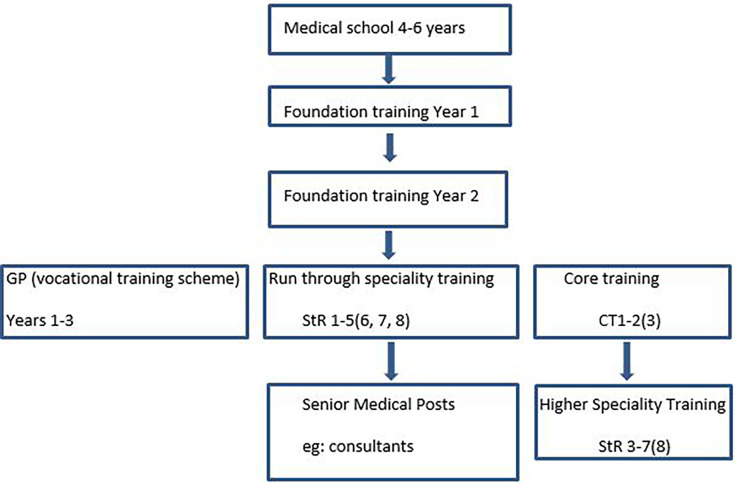
Overview of basic structure of medical eduction and training pathway.

Fig 1 provides an overview of the basic structure of medical education and training pathway in the UK. There is a common stem of training: medical school and foundation programme training, a 2 year broad based training programme that follows graduation from medical school. Successful completion of the foundation programme (FP) is an eligibility criterion for applying to speciality training. Cardiology is sub organised into core (ST1 and ST2) and higher speciality training (ST3 - ST7) (where the doctor has to apply for core and higher speciality training separately, and in total completes 7-8 years of training). The award of CCT (Certificate of completion of training) is required to work as a Consultant.

There is a mandatory requirement for Cardiology trainees to have cardiac device implant competencies to be signed off prior to completion of their training and commencement of their independent Consultant practice. This has traditionally been undertaken in an apprenticeship model with junior trainees initially assisting and then gradually transitioning to independent practitioners of these complex practical skills. The reduction in training hours compounded with reduction in procedures performed per operator has a perceived negative impact on this type of training.

Increasingly at every stage of training, learners see fewer patients, do less to them and as a consequence find themselves increasingly unprepared for practice ( Illing et al 2008).

In medical education, the triad of objectives, curriculum and assessment are paramount to structured education ( Crookston and Richter 2010). Once competencies have been established, specific learning objectives designed to help learners achieve competencies should be developed. This is an effective means of beginning with the “end in mind” and aligns itself, in my opinion, with strategic learning where in the process is tailored to gain the end result of a competent practitioner.

Medical careers have been characterised as a series of transitions ( Prince 2006). The transition from a pre-clinical to a clinical environment may result in the doctors feeling isolated and vulnerable ( Boehler et al 2004), as they find gaps in their knowledge and skills and have to restructure their cognitive processes as they move from theory to practice ( Prince et al, 2000; van Gessel et al 2003). This has further been hampered by the reduction in training time driven by European Working Time Directives ( EWTD 2009).

To this end simulation has been identified as a key strategic area to be developed within the North West healthcare workforce within the next five years ( HENW 2013). McCaughey and Traynor (2010) and Handley and Dodge (2013) further acknowledge the positive impact simulation has on student learning, particularly in developing students clinical skills, enhancing decision-making skills and closing the theory practice gap.

It is considered that deep, surface and strategic approaches to learning are considered essential theories to learning, with deep approaches favourable in enhancing positive learning experiences ( Lublin 2003) and potential impact upon patient care. The deep approach encourages students to become independent learners who take responsibility for their learning (Entwistle, 1988) and in doing so enables students to focus on underlying meanings, ideas and principles that can be transferred to other settings. This is crucial for an independent medical practitioner.

Rothgeb (2008) and Nagle et al (2009) advise that simulation encourages higher level skills linked to cognitive and effective learning experiences such as decision-making, critical thinking and communication. The primary aim of simulation is to improve patient safety and to help the student achieve competence, linking their theoretical knowledge with clinical practice ( Ganley & Palmer 2010; Ricketts 2011).

The reality however, is that for medics most education occurs through the care of real patients in clinical settings. Although various forms of simulation are becoming standard ( Issenberget al 2005; Cleland et al 2009), learning and assessment will occur predominantly in the clinical workspace for the foreseeable future.

It is therefore very important to explore the learner’s perspective before embarking on a change. After all, evaluation and the use of student feedback is identified as a significant part of the scholarship of teaching and learning ( Boyer 1990) and is important in respect of quality assurance and quality enhancement in the context of Higher Education (Biggs 2003). The inclusion of student perspectives has become a key Higher education policy initiative ( Browne, 2010).

It is with this in mind that the author undertook this study.

## Aims

This research project will use existing data derived from a national survey (questionnaire) previously undertaken (by the author) on Cardiology trainees on training opportunities, needs, attitudes and perceived barriers that exist within “current training” in cardiac devices.

This is intended to be Phase 1 of a multi-phased study.

Phase 2 will be guided by the results of this project and is likely to involve randomising Cardiology trainees to “standard training” versus metrics-based simulation training for cardiac device implantation to evaluate pre-defined outcome measures in both groups.

## Methods

The researcher is an Educational supervisor and is responsible for education and training of senior cardiology trainees including those ready to become consultants and take on independent practice. This provides her with insight into training opportunities and attitudes of trainers and trainees. She has been responsible for the survey undertaken previously that will be used for this research project.

When this project was conceived the researcher considered that it would involve a post-positivist approach rather than a pure positivist approach. The post positivist philosophy values the generalisation of findings beyond the particular study sample ( Schumacher and Gortner 1992), but considers them context dependant. Phillips and Burbles (2000) state that post positivisim rejects the view that knowledge is erected on absolutely secure foundations and accept fallibilism as an unavoidable fact of life. Individuals involved in a study are distinct and therefore generalisations are considered to be normative and predictive but not necessarily for each individual ( Charney 1996).

This is particularly true of a study of this nature where the participants are from different regions and regional variations in training are the norm rather than the exception.

The researcher’s involvement with education and role in the conception and design of the survey makes her position fallible. This is acknowledged at the very outset of the study.

Post positivists believe that we are all biased by our individual experiences and can never achieve total objectivity, but we can strive for it ( Trochim 2006). Upon reflection the researcher identifies with this particularly in the context of the study as the starting premise for this study was the fact that trainees voiced concerns about current training opportunities and the survey was generated in response to this.it would be impossible to eliminate bias.

To this end she believes that Phase 1 will have an inductive research strategy. Blaikie (2007) describes this as the collection of data, followed by data analysis which then proceeds to derive generalisations using inductive logic.

Plano, Cresswell and Clark (2008) suggest an abductive approach to research, which fits with her intention to make this study the first phase of a multi phased study in the area of exploring training options in Cardiology. They suggest that an abductive approach can be used to further a process of enquiry that evaluates the results of prior inductions through their ability to predict the workability of future lines of behaviour.

## Participants and sample size

Although the design of the questionnaire is outside the scope of this project these factors were borne in mind to make the survey results as representative of the target population (i.e all Cardiology trainees) as possible.

The study was carried out in the UK.

The questionnaire ( Appendix 1) was sent to all Cardiology trainees by the British Junior Cardiologists Association (BJCA) with the intention of evaluating trainee perception of current training opportunities in cardiac device implantation. This is an independent body representing the junior trainee Cardiologists. This was done in an attempt to avoid coercion and/or bias.

The survey was sent out by survey monkey and a reminder sent 2 weeks later for non-respondents.

All potential participants were contacted by the BJCA via their work email and a covering letter (Appendix 2) sent to explain the rationale behind the survey with an attached link to the survey itself. Consent was implied in participation.

The questionnaire comprised of 6 questions of which the first was a free text question to establish context (stage of training) and the rest required a yes/no (dichotomous) response with space for free text.

The data collected will include the following variables (as defined in the survey):

• Year of training (free text)
• Need to trouble shoot cardiac devices (Y/N)
• Need to reprogram cardiac devices (Y /N)
• Feels competent to re program (Y/N)
• Opportunity for implant ( Y/N)
• Felt competent implanting (Y/N)

## Design

This research will employ a mixed qualitative and (semi) quantitative exploratory design method of enquiry as it involves interpretation of responses to the survey from cardiology trainees. In keeping with the phased nature of the bigger project - Phase 1 will concentrate on qualitative methodology which is expected to be hypothesis generating, and interpretive whilst studying a complex environment and answering the complex “why” question.

## Data Collection and analysis

As mentioned earlier, no personal interaction with the trainees was required, and the study was envisaged after the data had been collected for educational purposes previously. This meant that the researcher had no effect on the data outcome.

Each respondent was anonymised and given a number as an identifier, which enabled alignment and analysis of the results.

The researcher then identified useful quotes from the free text, coding segments of information into broad themes. With the configuration of the qualitative ‘persuasive’ data, the researcher will use this as the ‘window’ to the ‘mind’ of the trainee Cardiologist ( Parahoo 2014) and explore themes that may explain the trends in current training. The “free text” will be broken down into categories and these categories will then be grouped into manageable themes to make sense of it (Arride-Sterling 2001).

Because the questionnaire is measuring subjective competence, and there are no adequate indicators of actual competence to indicate actual competence, no analysis of predictive or concurrent validity is possible.

## Validation analysis

To ensure the construct validity of the questionnaire, the proportions of missing data per items were examined (high levels of missing data suggesting that an item is inappropriate or unclear).

Research Ethics Committee (Edgehill) approval was obtained prior to commencement of this project.

## Results

123 responses out of a maximum possible 800 (National Training Numbers in Cardiology) i.e 15% sample size were received. There was no missing data on any of the returned questionnaires.

**Fig 2.**
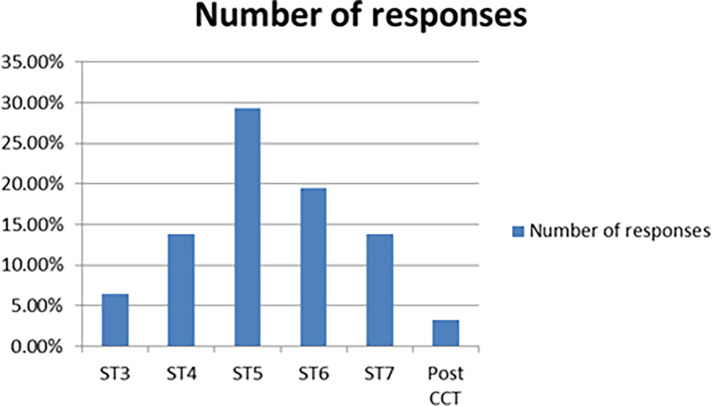
Response rates and stage of training.

The respondents were from varying stages of training ranging from ST3 to ST7 and post completion of certificate of training. The details are shown in Fig 2.

Cardiac device training has 2 main components - implantation of the device itself which includes surgical skills and follow up of these patients which is more clinic and out-patient based and includes dealing with issues arising from the cardiac device itself.

In the past 6 months over 45% of them had encountered a problem with a cardiac device (ranging from 45 to 90%) and over 32% (32-78%) of them depending on the device had been asked to trouble shoot. The details are shown in Fig 3a and 3b

58/123 (47%) offered further comments on training. 50% of them were not happy with current training.

**Fig 4.**
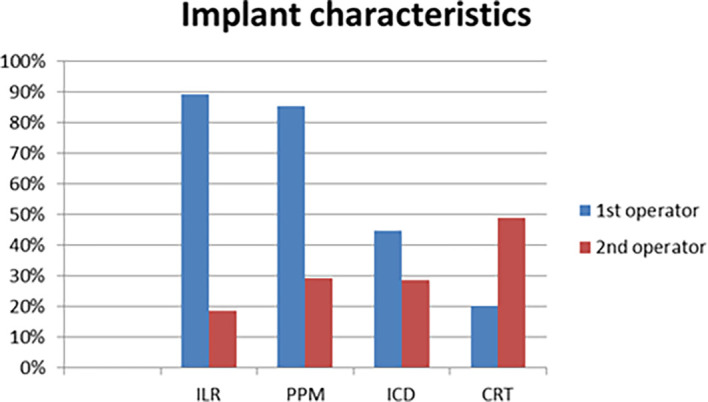
Implant characteristics based on complexity of device.

Opportunity for implant varied depending on the complexity of the procedure from 90% of respondents having had the opportunity to implant a loop recorder (simplest of devices) as a first operator to 20% for a Cardiac Resynchronisation Pacemaker (the most complex of devices). The details are illustrated in Fig 4.

**Fig 5.**
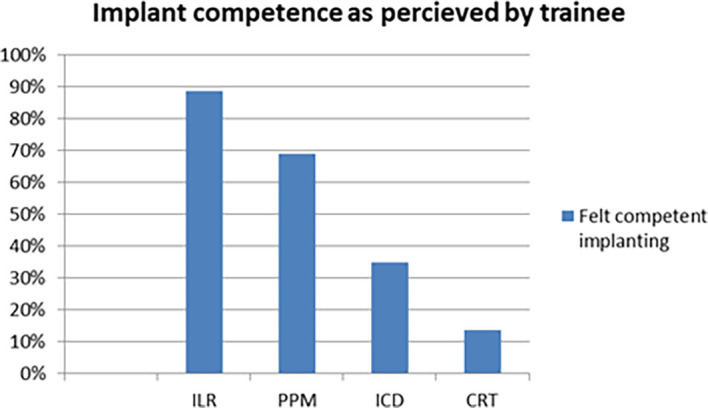
Perceived implant competence with complexity of device.

The perception of competence also varied depending on complexity of implant from 89% feeling competent with a loop recorder to only 14% feeling competent with a Cardiac Resynchronisation Pacemaker ( Fig 5).

## Qualitative analysis

Phenomenological analysis ( Sullivan 2012) was attempted with the free text with the identification of themes, meaning units and a “lived world experience”. Groups of words with similar meaning defined a category and theses were then grouped into manageable themes.

The emerging themes were broadly of “Opportunity” and “Attitude”. The implantation training was hampered by opportunity in terms of number of implants, access to implants and poorly structured training. Exploring these themes further highlighted that the trainee’s time was heavily subscribed and committed to other service provision leaving them less time than they would have liked to focus on hands on training in theatre or in the dedicated clinics doing programming.

The “attitude” commented on was one of lack of interest and involvement on part of the “trainer”. Time pressures and other commitments seemed to contribute to this but there was an underlying feeling of training NOT being a priority.

Service commitment was cited numerous times as a possible reason for inadequate training due to reduced opportunities as well as a negative impact on attitudes to training.

The table below summarises this-

**Table 1.** Emerging themes from responses

OPPORTUNITY	ATTITUDES
**Implants** Inadequate, hardly, not enough exposure , not enough time , rota commitments , sporadic, “adhoc” **Programming** No exposure, No training , Busy doing other things, inadequate, sporadic	**Trainer** Not a priority , Badly done, Badly taught, no focus on training, not confidence, negative, lack of commitment **Trainee** Loss of confidence, loss of interest, undermined, frustrated
**Service Commitment** **Competence based training not helping**

Interestingly, Competence based training was cited as hindrance rather than a positive in terms of training - specifically the requisite “level 2” sign off meant that trainees didn’t actually need to be independent in pacemaker implantation making this a low priority and a barrier to training opportunity.

## Discussion

An online survey has methodological limitations but was the only practical way to reach a large number of trainees spread across the country. Our sample was heterogeneous and included Cardiology registrars at different stages of training (from first year to final year and beyond) and across various regions, yet we found few differences related to stage of training or region of training.

This is surprising as one might expect that the registrar’s confidence grows as they progress through training and might be a reflection of the fact that less than 15% of respondents were senior trainees (ST7 and above). It is possible that this is responder bias i.e those satisfied with training and were confident and competent did not bother with the questionnaire.

Physician surveys are an important tool in health services and policy research, providing cost effective sources of information on physician’s attitudes, knowledge and practices related to care delivery and training ( Mosca et al 2005). Despite their importance however physician surveys are characterised by their low response rates, raising concerns about their validity and generalizability of their findings ( Kellerman and Herold 2001). Specifically low response rates raise concerns about non response bias or the likelihood that non responding physicians will be systematically different from the population understudy. This concern is supported by research showing modest differences between responders and non-responders and between early and late respondents on demography and/or practice -related characteristics ( Stocks and Gunnel 2000).

A sample size of 30 is usually the accepted minimum if quantitative analysis is to be performed ( Cohen et al 2011; Denscombe 2010). Of course, the validity of the sample size is based on much more than the perceived minimum, it is also based on the size of the population being sampled. Creswell (2012) defines the population of a study as ‘a group of individuals who have the same characteristics’.

In our study, 123 trainees responded to the survey which equates to a 15% (of total possible responses). In the researcher’s opinion this is a reasonable representation of the target population and attributes this to competing time pressures on the trainee. She is however, mindful of the possibility that the non-respondents were fully satisfied with current training provision and therefore did not feel the need to respond to the questionnaire resulting in response rate bias. There did not appear to be a regional variation (as volunteered by the respondents) in the views expressed, making this unlikely as this was a national survey and provided us with an overview of training from an individual perspective.

Previous qualitative research by Kasprzyk et al (2001) mention questionnaire length as a factor in willingness to participate. Furthermore, Sudman (1985) recommended the need to establish relevance (by means of an accompanying cover letter for instance) as a means of improving response. Interestingly endorsements by local, state or national organisations typically improved physician participation ( Asch and Christakis 1994).

The author was mindful of this in the design of the study and questionnaire was brief and contextualised with the use of an introductory letter citing the purpose of the questionnaire.

Successful strategies for high recruitment in healthcare surveys include initial face to face recruitment, persistent reminders( including follow up phone calls) and contact with high level decision makers ( Elkins et al 2011). These strategies were not used in our study so as to avoid breach of confidentiality. Particularly, no demographic details were included in the study so as to protect the identities of the respondents. An email reminder was sent to encourage response. The use of high level decision makers (i.e training programme directors) was deliberately not made as whilst it might have improved response rates the author felt there would be a significant conflict of interest and resulting bias in the trainee responses.

External validity will be emphasised with the need for the natural environment to be left unmanipulated ( Guba 1990). Within the context of this project the responses were anonymised (unless participants chose to disclose their identity) and no incentives were offered to participants (training or otherwise).

Robson (2002) advocates a good flexible research design that comprises rigorous data collection and analysis and recognition of the possibility of multiple realities. It recognises the researcher as an instrument of data collection with particular focus on participant views. The author identifies with this view and has made an attempt to keep the focus on the trainee’s overall perspective with careful attention to the “free text” provided by the trainees.

Mckenna (1997) suggests that whilst questionnaire surveys may provide measurement scales for measuring phenomenon qualitative research may be more appropriate for gaining an understanding of people’s perceptions and understanding which may be less amenable to measurement.

The questionnaire in this study highlighted some important findings however, it is the “soft free text” that contextualises the findings and informs the researcher of next steps and the real issues with current training. Grypdonck (2006) noted that that researchers can only understand perception and behaviour from participants own perspectives in their own words and in the context in which they exist. This is particularly important in a study like this where regional variation in training would influence the outcomes of the study as would the stage of training and mind set of the trainee at the time of responding to the questionnaire.for instance if the trainee had has a bad week with on call commitments interfering with dedicated training time it is likely that this would have impacted negatively on his responses to the questions.

The researcher also is mindful of a quantitative method such as a survey questionnaire being open to subjectivity of the researcher and the study participants ( Yates 2002) but firmly believes that this adds to the value of the study.

Interestingly the combination of research methods has been perceived as important in the evaluation and assessment of increasingly complex health care interventions and system ( Kinn and Curzio 2006) and that is exactly what the author has attempted to do.

Cole and Eyles (1997) propose three roles for qualitative data and analysis in the Health Impact Assessment (HIA) process. These roles reflect the ways in which qualitative data ‘support or augment’ quantitative impact assessments as a precursor to quantitative work to generate a hypothesis.

The second role accredited to qualitative inquiry is predicated on the possibility of ‘parallel analysis which actively integrates qualitative and quantitative. Baum (1995) contends that researchers may select from ‘a smorgasbord of methods’. Douglas et al (2001) suggest that the different data types are useful in providing ‘different perspectives’ on the same issues.

The third role is understanding of the relationships that persist between certain possible objects of inquiry. This refers to a secondary activity performed by qualitative analysis. In this instance, the objects have already been examined in themselves (presumably by either parallel analysis or purely quantitative means) i.e issues with training, type of training and potential shortfalls (identified by the structured questions)., but now qualitative analysis is utilized to provide a less abstract and more situated context for the data to understand “why” (free text)..

The author identifies with this aspect of qualitative data and in her opinion the most useful information gleaned from this survey was from the “free text” volunteered by the trainees highlighting the main issues with current training. This helped understand the reasons for the poor training and lack of exposure to requisite skills.

The results of this survey suggest that training was perceived as inadequate by the vast majority of respondents and this in turn reflected on their ability to perform the tasks expected of them resulting in loss of confidence and frustration.

The emerging themes were one of opportunity and attitude. Exploring “opportunity” (lack of) further highlighted the main reasons to include inadequate number of implants, inexperienced operators, inadequate sessions in theatre, reduced exposure time. The opportunity to do the very simple cases existed but rapidly declined with the increasing complexity of cases. This directly translated into a progressive reduction in confidence with increase in complexity of cases.

Interestingly, the issue with inadequate numbers was not just for trainees but also for “trainers” resulting in them keeping the operating for themselves (further depriving the trainee of the chance to increase his numbers and confidence). When we looked at “attitude” the trainees perceived trainer attitude as disinterested and disengaged. A few trainees felt the “trainers” were under confident and this reflected in their poor attitude to training. An underlying theme of “service commitment” seemed to run through the comments and was offered as the reason for the loss of training.i.e too busy supporting the running of the hospital to make training a priority. The conflict between “training” and “service” time was highlighted by many respondents citing on call followed by forced rest times as a reason for missed training opportunities.

Worryingly, some trainees cited Competency Based Training (CBT) as a reason for inadequate training with trainers using the formative assessments to avoid training rather than enhance it. A few trainees suggested that the requisite number of directly observed procedural skills (DOPS) were obtained for sign off with little focus on the true ability of the trainee.

Competency-based medical education (CBME), by definition, necessitates a robust and multifaceted assessment system ( Norcini et al 2008). ( Hawkins & Holmboe 2008) mention effective assessment provides the information and judgment necessary to enable program-level decisions about trainee advancement to be made reliably and fairly. There is also a suggestion that this sort of competency based assessment may reduce training time ( Carraccio et al 2002). This is worrying given the perceived shortfall in training opportunities highlighted by this survey.

( Nelson et al 2007) define a clinical microsystem as a small group of people who work together on a regular basis to provide care to patients. It has clinical and business aims, linked processes, and a shared information environment, and produces performance outcomes. These microsystems provide the context for training and assessment and akin to the environment that the Cardiology trainees work in. It is important to consider how the culture and functionality of these microsystems affect assessment and trainee outcomes in the long term ( Rethans et al 2002). The survey highlights the “non-training” culture within this environment with service provision taking priority over training.

A simulation based instruction strategy provides a safe, risk-free yet powerful approach to medical education (Ziv et al 2003). Studies have demonstrated the effectiveness of simulation based training in undergraduate medical education ( Weller 2004)( Fitch2007).

## Strengths and weakness

The questionnaire was sent to all national trainees and we had a reasonable number of responses from national trainees giving an overall feel for training at a speciality level in Cardiology. In UK the curricula are nationally set through the Royal Colleges and GMC and it is important that the whole perspective for training is explored to institute changes in the future and improve training.

While the perception of inadequate training may not be indicative of ability it does have a potential effect on the doctors confidence to take on or even avoid certain aspects of the role. There is the potential for bias in response rates with the more satisfied trainees not responding to the questionnaire

## Conclusion

The current training for cardiac devices lacks structure and issues with opportunity for hands on experience. This lends itself to the use of simulated learning with dedicated sessions and appropriate and timely work based assessment. The author hopes to explore this option by designing phase 2 of the study by randomising Cardiology trainees to “standard training” versus metrics- based simulation training for cardiac device implantation to evaluate pre-defined outcome measures in both groups.

## Take Home Messages

**Figure:**
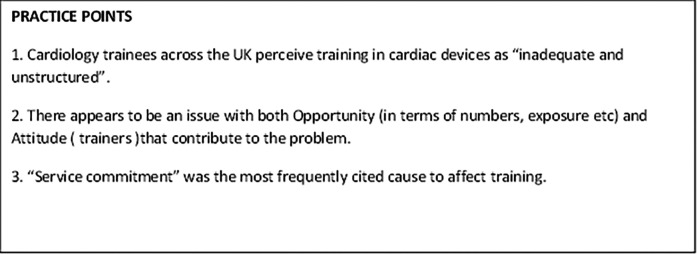


## Notes On Contributors

Dr Rao is a Consultant Cardiologist in Heart Failure and Cardiac Devices. She has a strong interest in training and education and is involved with curriculum design and execution in undergraduate and post graduate medical education.

Dr Lee is a trainee doctor currently doing his core medical training in the Northwest.

The authors report no declarations of interest.

## Figures and Tables

**Fig 3a.**
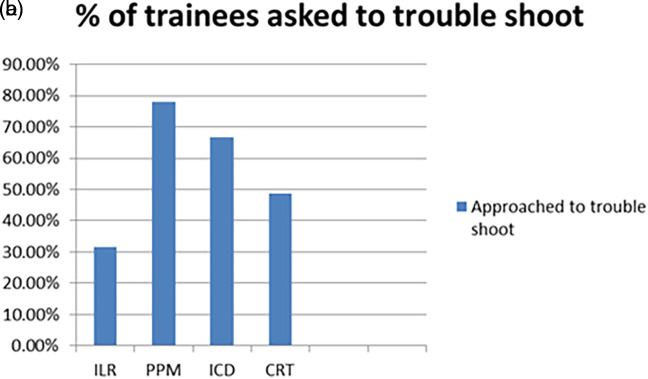
Need to troubleshoot devices.

**Fig 3b.**
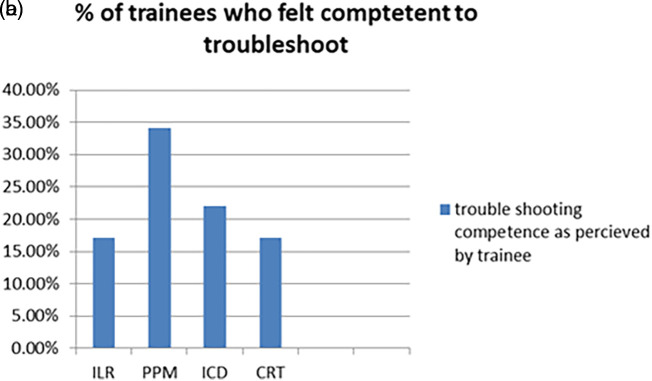
Trainee perceived competence in trouble shooting.
