# Supplementary material for: “Current training” in Cardiac devices – a Cardiology trainee perspective: a questionnaire survey
Source: MedEdPublish (2016). 2017 Jul 17;6:130. [Version 1] doi: 10.15694/mep.2017.000130 (PMC10885280; doi:10.15694/mep.2017.000130)
Supplement: Peer review history

# Reviewer report: Reviewer response for version 1

**DOI:** 10.21956/mep.19315.r28186

**Published:** 2017-07-24

**Authors:** Trudie Roberts

**Affiliations:** 1 Leeds Institute of Medical Education

This review has been migrated. The reviewer awarded 4 stars out of 5 An interesting paper which like Trevor Gibbs made me reflect on the increasing 'technolisation' of medicine in general and how this is or probably is not covered in PG curricula. As the lessening of importance of basic clinical skills such as auscultation in cardiology for example there will need to be a review of modern training needs. Sadly college exams will probably hold this back

Reviewer Expertise:

NA

No decision status is available

# Reviewer report: Reviewer response for version 1

**DOI:** 10.21956/mep.19315.r28185

**Published:** 2017-07-22

**Authors:** Michael SH Wan

**Affiliations:** 1 University of Notre Dame

This review has been migrated. The reviewer awarded 4 stars out of 5 An important paper reviewing the trainees' perception of device implantation and trouble-shooting competencies. This highlighted the general inadequacy of training in this aspect. The phase 2 study would be very useful to investigate whether a more structured program with simulation would improve this. It is clearly expected that both exposure and opportunity to implant CRT devices would be more difficult especially as the first operator. One of the major factor would be the busy commitments of the senior physicians and consultants resulting in less time for teaching and skills training. "Protected time" for training would be another aspect that could help.Those non-responders to the survey could be too busy or too frustrated to respond.

Reviewer Expertise:

NA

No decision status is available

# Reviewer report: Reviewer response for version 1

**DOI:** 10.21956/mep.19315.r28184

**Published:** 2017-07-18

**Authors:** Trevor Gibbs

**Affiliations:** 1 AMEE

This review has been migrated. The reviewer awarded 4 stars out of 5 An interesting and well thought through paper that highlights an inadequacy in postgraduate training. Although related to cardiology specifically, I feel that these issues can be easily applied to other specialist training programmes that are a mixture of clinical and technological expertise- and an issue that will probably increase in importance as technology continues to play an important and growing element of healthcare.I am happy to suggest that this is an important paper for all those concerned with higher postgraduate training and although it is very UK-centric there is little to suggest that it doesn't apply to other areas of the world.My one concern is the low number of responses. The authors suggest that this maybe related to the apparent satisfaction of those not responding - I would suggest that it also may reflect dissatisfaction with either training or the system and perhaps this needs to be investigated as a separate entity to this piece of research

Reviewer Expertise:

NA

No decision status is available
